# Does Mother–Child Interaction Mediate the Relation Between Maternal Depressive Symptoms and Children’s Mental Health Problems?

**DOI:** 10.1007/s10826-015-0309-1

**Published:** 2015-10-27

**Authors:** Marleen M. E. M. van Doorn, Rowella C. W. M. Kuijpers, Anna Lichtwarck-Aschoff, Denise Bodden, Mélou Jansen, Isabela Granic

**Affiliations:** Behavioural Science Institute, Radboud University Nijmegen, P.O. Box 9104, 6500 HE Nijmegen, The Netherlands; Pro Persona Youth (Netherlands Institute of Mental Health), Arnhem, The Netherlands

**Keywords:** Maternal depression, Childhood depression, Mother–child interaction, Observations, Internalizing and externalizing mental health problems

## Abstract

The relation between maternal depressive symptoms and children’s mental health problems has been well established. However, prior studies have predominantly focused on maternal reports of children’s mental health problems and on parenting behavior, as a broad and unilateral concept. This cross-sectional study examined specific observed mother–child interaction behaviors through which maternal depressive symptoms are assumed to affect children’s mental health problems. We expected higher rates of maternal depressive symptoms to predict higher rates of children’s mental health problems, and we expected this relation to be mediated by low maternal warmth and high maternal psychological control. The sample consisted of 111 mother–child dyads referred for treatment. The mother–child interaction behaviors were coded according to the observed mother–child interaction tasks. Children’s mental health problems were assessed using both maternal reports and children’s self-reports. As expected, the results showed that maternal depressive symptoms were strongly related to maternal reports of children’s internalizing and externalizing mental health problems. Surprisingly, maternal depressive symptoms were unrelated to children’s self-reported depressive symptoms. Furthermore, mother–child interactions did not mediate the relation between maternal depressive symptoms and child mental health problems. Maternal depressive symptoms were associated with high maternal warmth, and high psychological control was associated with high levels of mother-reported externalizing mental health problems in children. These results partially replicate previous findings but add to these by using observational methods and multi-informant data. The importance of using a multi-informant and multi-method approach in assessing children’s mental health problems in clinical practice and research are discussed.

## Introduction

The lifetime prevalence rate of depression ranges from 8 to 12 % (Andrade et al. 2003). According to the World Health Organization (2008), women are not only twice as vulnerable to depression compared to men, but also experience 50 % more disease burden compared to men. Additionally, women with children have an increased risk of experiencing more depressive symptoms compared to women without children (Kessler 2006). These findings are worrisome, since research has shown that children of mothers with depressive symptoms are at a higher risk for poor psychosocial development, such as low self-esteem, negative attribution styles, heightened emotionality, and negative affect. They are also more likely to experience social and achievement problems and to suffer from mental health problems, such as depressive or anxious symptoms and behavioral disorders (Goodman and Tully 2006; Hammen et al. 2003). While the adverse effects of maternal depressive symptoms on child development are well documented, less is known about the underlying mechanisms that mediate the transmission of risk (Goodman et al. 2011). A well-documented mechanism is mother–child interaction behavior, but up until now, studies have mainly used maternal self-report to assess both mother–child interaction behavior and mental health problems in children, calling into question the extent to which these relations should be attributed to reporter bias in mothers with depressive symptoms (Kraemer et al. 2003). Additionally, studies investigating the mediating effect of observed mother–child interaction behaviors have examined mother–child interaction as a broad concept (e.g., negative vs. positive), and did not focus on specific types of interaction behaviors (Burt et al. 2005).

Goodman and Gotlib (1999) proposed an integrative model of the transmission of risk for children of mothers with depressive symptoms, identifying the following mechanisms: (1) heritability of depression, (2) dysfunctional neuroregulatory mechanisms, (3) exposure to negative maternal cognitions, behaviors, and affect, and (4) the stressful context of the children’s lives. Mother–child interaction is part of the third mechanism of this integrative model and entails several components and processes. The first component within this domain states that maternal depressive symptoms are expressed by negative cognitions, behaviors, and affect. Adults with depressive symptoms are more likely to endorse negatively biased self-perceptions and cognitions (Gotlib and Neubauer 2000). In particular, mothers with depressive symptoms have been found to have a negative perception of their role as a parent (Goodman et al. 1993), to experience more helplessness regarding their children’s development, and to view themselves as less capable of influencing their children in a positive manner (Kochanska et al. 1987). Mothers with depressive symptoms were also found to expose their children to depressive behaviors and affect. They showed increased negativity (e.g., intrusiveness, control, hostility), greater disengagement (e.g., ignoring, withdrawal, silence), and less positivity (e.g., warmth, praise, affection) in interaction with their child compared to non-depressed mothers (Lovejoy et al. 2000). Furthermore, mothers with depressive symptoms showed more anger, sadness, and irritable affect towards their child (Conn et al. 1990).

The second component suggests that because of these negative cognitions, behaviors, and affects, mothers experience difficulties interacting with their children and fail to meet their social and emotional needs. According to the attachment theory, children develop an attachment relationship with their primary caregiver who provides the child with an internal working model that encourages the child to explore the world and to regulate his or her feelings effectively (Bowlby 1982). Children of mothers with depressive symptoms had higher rates of insecure attachment (Martins and Gaffan 2000). In turn, higher rates of insecure attachment in the child were associated with higher rates of mental health problems in the child (Brumariu and Kerns 2010). Parental sensitivity seems to be crucial in forming attachment representations. Insensitivity and unresponsiveness in the mother–child interaction were linked to insecure attachment (Egeland and Farber 1984). In addition, psychological control can also lead to an insecure attachment. Children who grew up in an inconsistent or psychologically controlling parenting environment were likely to experience feelings of insecurity and dissociation (Soenens et al. 2008), which might lead to insecure working models and eventually to more mental health problems.

The third component describes that this problematic interaction with the mother impairs the development of adequate social skills and cognitive styles in children. Children of mothers with depressive symptoms were rated as less popular by their teachers compared to children of mothers without depressive symptoms (Goodman et al. 1993). Furthermore, Sroufe et al. (2005) showed that insecurely attached children displayed more negative affect in interaction with other children, presented less prosocial behavior, and reacted more aggressive compared to securely attached children. The fourth and last component postulates that children express cognitions, behaviors, and affect similar to their mother, which they attain through social learning processes, such as modeling. Children of mothers with depressive symptoms displayed more negative cognitions (i.e., lower self-concepts, more self-criticism, less positive self-descriptive adjectives) and more negative affect and behavior (i.e., less responsiveness, less activity, less content, flatter affect) compared to children of mothers without depressive symptoms (Dawson et al. 1997; Garber and Robinson 1997). Goodman and Gotlib (1999) argued that children’s deficient social skills and cognitive styles, in addition to their attained negative cognitions, behaviors, and affect, would eventually put them at risk for developing mental health problems. It is therefore crucial to gain a more detailed understanding of the interaction between mother and child regarding the transmission of maternal depressive symptoms to children’s mental health problems.

Recent studies have found evidence for a mediating effect of typical mother–child interaction behaviors, such as neglectfulness, positive, and negative affect, positive relations, and disciplinary practices, on the relation between maternal depressive symptoms and children’s mental health problems, both internalizing and externalizing (Karazsia and Wildman 2009; Kiernan and Huerta 2008; Pugh and Farrell 2012). Although these mediation studies have made an important contribution to the understanding of the link between maternal depressive symptoms and children’s mental health problems, they are subject to limitations. One important limitation is the reliance on maternal report regarding both children’s mental health problems and mother–child interaction behavior (Karazsia and Wildman 2009; Kiernan and Huerta 2008). This may cause potential reporter bias, since cognitive theories of depression suggest that mothers with higher levels of depressive symptoms seem to perceive various aspects of their life, including their child’s mental health, in a more negative way compared to mothers with lower levels of depressive symptoms (Kraemer et al. 2003). To prevent possible reporter bias regarding the nature and quality of mother–child interaction behaviors, observation of mother–child interaction behaviors might be preferred to self-report in studies examining mothers with depressive symptoms. However, observation studies with a specific focus on maternal depressive symptoms and mediating mother–child interactions are scarce. To our knowledge, only one observational study investigated the mediating effect of observed mother–child interaction behavior on the relation between maternal depressive symptoms and children’s mental health. Burt et al. (2005) found that negative mother–child interaction partially mediated the association between maternal depressive symptoms and adolescent mental health problems in male adolescents only. The authors focused on global, overarching codes of mother–child interaction behavior (e.g., negative vs. positive); therefore, it could not provide information about specific mother–child interaction behaviors that may be most indicative of depressive mothers’ parenting and may have direct implications for prevention and intervention efforts.

Empirical literature on specific mother–child interactions identified maternal warmth and maternal control as crucial factors in explaining children’s internalizing and externalizing mental health problems (Albrecht et al. 2007; Casas et al. 2006; McNamara et al. 2010). Recent meta-analyses confirmed that less maternal warmth and more maternal control were related to increased children’s mental health problems (Kawabata et al. 2011; McLeod et al. 2007). However, the construct control has been criticized for the inability to differentiate between behavioral and psychological control (Soenens and Vansteenkiste 2010). Behavioral control is defined as the ability of the parent to regulate the behavior of the child by using discipline (providing rewards and punishments) and monitoring. Psychological control is the ability of the parent to hinder independence and autonomy of the child by using emotional manipulation (e.g., guilt or shame inducing, love withdrawal), constrain, and invalidation (Ballash et al. 2006; Barber et al. 2005). Moderate levels of behavioral control have been related to positive outcomes, whereas psychological control has been associated with children’s internalizing and externalizing mental health problems (Barber et al. 2005).

The current study was designed to examine the mediating effect of mother–child interaction on the relation between maternal depressive symptoms and children’s mental health problems. We addressed several gaps in the prior empirical literature. First, to bypass any possible reporter bias, we used observations of the mother–child interactions instead of maternal report, and we included both mother-report as well as children’s self-reports of children’s mental health problems. Second, to extend past observational research that focused on broad, global categories of positive and negative interaction patterns as a mediator, we focused on maternal warmth and maternal psychological control, two more specific mother–child interactions that are known to be related to both maternal depression as well as child mental health. The main objective of the present study was to examine observed maternal warmth and maternal psychological control as mediators in the relationship between maternal depressive symptoms and children’s mental health problems, as reported by both mothers and children themselves. We expected that higher rates of maternal depressive symptoms would predict higher rates of mother-reported internalizing problems and externalizing problems of children as well as higher rates of child self-reported depressive symptoms. Furthermore, we hypothesized that low maternal warmth and high maternal psychological control will mediate these relations.

## Method

### Participants

This study was part of a larger treatment study on aggressive children that aimed to determine the processes of change related to treatment success (Granic et al. 2007). The parents of all children with aggressive behavior that were referred to one of two participating Canadian mental health agencies by a mental health professional, teacher, or parents themselves were informed about the study and asked to participate. They were reassured that declining to participate would not affect their further treatment. If they participated in the research, they were offered CAD$10.00. The inclusion criteria of the large treatment study on aggressive children were a clinical or borderline-clinical score (T ≥ 65) on the externalizing subscale of the parent-report form of the Child Behavior Checklist (CBCL; Achenbach 1991) and sufficient knowledge of the English language to complete the questionnaires without an interpreter. The child also had to live with the mother (biological, step-, or adoptive). Children were excluded if they were diagnosed with a pervasive developmental disorder or if they had an IQ below 70. Information about other clinical diagnoses was not available. Only the data from pre-treatment assessments were used in the current study and only mother–child dyads that had complete data on all of the study variables at pre-treatment were included. Initially, 199 children between 8 and 12 years of age and their mothers consented to participate. Of the original sample that consented to participate, 88 dyads (44 %) had to be excluded due to missing data (refusing to be videotaped, not showing up at the research appointment, or not filling in some of the questionnaires). Unfortunately, families with aggressive children who visit outpatient clinics for treatment generally have high dropout rate (e.g., Prinz and Miller 1994). The final sample for the present study consisted of 111 dyads. The children ranged in age from 8 to 12 years (*M* = 9.38, *SD* = 1.15) and 88 % were boys. Most children (37 %) resided in intact families, 33 % lived in single-parent (exclusively maternal) households, 18 % in blended families (e.g., living with biological mother and stepfather), 7 % lived with adoptive parents, and 5 % in other family compositions (e.g., mother and child living with grandparents). Children were mostly Caucasian (82 %), followed by African-American or Caribbean (12 %), Latin-American (2 %), and other ethnical backgrounds (e.g., Asian) (5 %).

The mothers ranged in age from 26 to 56 years (*M* = 39.15, *SD* = 6.47). Most mothers (46 %) was married, 19 % were single (never married), 18 % were divorced or separated, 14 % lived in a common law relationship, and 3 % reported another relationship status (e.g., widowed). Maternal education level was relatively high, with 8 % having a post graduate or professional degree. Furthermore, 49 % graduated from community college or university, 29 % graduated from high school, 9 % attended high school but did not graduate, and 5 % finished grade 8 or less or had other forms of education. Family income was relatively high as well, 46 % made over $60,000, 19 % made $40,000 to $59,000, 18 % made between $20,000 and $39,000, and 18 % made under $20,000 per year.

The attrition analyses showed no differences between the total (*N* = 199) and final (*N* = 111) sample in maternal depressive symptoms *t*(190) = 1.64*, ns;* internalizing problems *t*(195) = 0.79, *ns*; externalizing problems *t*(195) = 0.54, *ns*; maternal warmth *t*(193) = −0.65, *ns*; maternal psychological control *t*(153) = −1.75, *ns*; maternal age *t*(175) = −1.12, *ns*; maternal education *t*(193) = 1.37, *ns*; and family income *t*(187) = −1.52, *ns*. Furthermore, no differences emerged between the two samples on maternal relationship status χ^2^ (6, *N* = 198) = 3.18, *ns*; residence of the child χ^2^ (6, *N* = 199) = 7.94, *ns*; and ethnicity of the child χ^2^ (5, *N* = 197) = 3.18, *ns*. The final sample differed from the original sample only in gender of the child χ^2^ (1, *N* = 199) = 7.85, *p* < .01. The percentage of boys in the final sample (88 %) was higher compared to the original sample (81 %).

### Procedure

The data collection took place before the start of the treatment and included a home visit by a research assistant where families completed questionnaires. First, mothers and children were asked to complete the consent forms and a modified version of the Issues Checklist (Robin and Weiss 1980), which lists a number of potential sources of conflict between parents and children (e.g., bed time, lying, swearing). Next, mother and child were placed together (e.g., at a kitchen table, on a couch) and asked to engage in three separate discussions. The first and third discussion lasted 4 min and contained a positive, hypothetical topic, such as winning the lottery or planning a trip together. These topics were randomly assigned and counterbalanced across participants. The second discussion on a conflict topic chosen from the previously completed Issues Checklist lasted 6 min. Based on the procedure of Forgatch et al. (1985), this entailed asking mother and child separately to report whether they had argued about each issue in the past 2 weeks, to report the frequency and intensity of these discussions (on a 5-point scale from calm to angry), and to report whether the issue had been resolved. The hottest unresolved topic (as indicated by both mother and child) was chosen for the conflict discussion (e.g., going to bed on time, fighting with sibling). The research assistant gave instructions before each of the three discussions and then left the room. The interactions were recorded on a digital video camera. After the discussion tasks, mothers completed the measures of the child’s mental health problems and of their own depressive symptomatology. Children completed a questionnaire about their own depressive symptoms.

### Measures

#### Beck Depression Inventory (BDI-II)

Maternal depressive symptoms were measured using the beck depression inventory second edition (BDI-II; Beck et al. 1996). The BDI-II measures depressive symptomatology in 21 items and shows high validity in differentiating depressed from non-depressed individuals (Richter et al. 1998). Items are rated on a 4-point scale ranging from 0 to 3 in terms of intensity of symptoms (e.g., change in appetite) and attitudes (e.g., pessimism) during the past 2 weeks. Scores of 0–9 on the BDI-II indicate the non-clinical range (no signs of depression) and scores of 10 and above indicate the clinical range (mild to severe signs of depression). Reliability of the BDI-II scale was excellent (Cronbach’s α = .92).

#### Child Behavior Checklist (CBCL)

Children’s mental health problems were measured using the (CBCL; Achenbach 1991). The CBCL is a widely used parent-report questionnaire that has two broadband factors, internalizing (anxious, depressed and withdrawn behavior) and externalizing mental health problems (aggressive and hyperactive behavior). Mothers were asked to rate each of the 113 items on a 3-point scale ranging from 0 (does not apply to the child) to 2 (clearly or often). T-scores of 64 or less represent normal range, T-scores of 65–69 indicate borderline-clinical range, and T-scores of 70 or higher represent the clinical range. The psychometric properties of the CBCL have been well established (Ivanova et al. 2007).

#### Short Mood and Feeling Questionnaire (SMFQ)

Children’s self-reported depressive symptoms were assessed using the Short Mood and Feeling Questionnaire (SMFQ; Angold et al. 1995). The SMFQ is a self-report questionnaire that measures symptoms of depressive disorders in children and adolescents. Children are asked to rate each of the 13 items on a 3-point scale ranging from 0 to 2 in terms of intensity of depressive symptoms during the past 2 weeks (e.g., “I felt miserable or unhappy”, “I did everything wrong”). A score of 8 or higher suggests the clinical range (Angold et al. 1995). Various studies have demonstrated satisfactory psychometric properties of the SMFQ. The SMFQ showed good reliability (Angold et al. 1995), convergent validity (Wood et al. 1995), construct validity (Sharp et al. 2006), and criterion validity (Angold et al. 1995). In the current study, the SMFQ showed good reliability (Cronbach’s α = .87).

### Coding

The video recordings of the conflict discussion task were used to assess maternal warmth and maternal psychological control. The coding system was based on prior work of several researchers (Ballash et al. 2006; Barber 1996; Eyberg et al. 2005; Greco and Morris 2002; McLeod et al. 2007; Siqueland et al. 1996; Soenens and Vansteenkiste 2010). The coding system contained 27 items on a 9-point scale ranging from 1 (not at all) to 9 (very much), indicating the extent to which the behavior was present during the discussion task. We decided to delete 4 items that did not measure maternal warmth or psychological control and 7 items that were almost non-existent in the sample [>90 % had a score of 1 (‘not at all’) on the item]. Due to the small sample size, a proper confirmatory factor analysis could not be conducted. However, the results of an initial CFA, which utilized a WLSMV-estimator (i.e., Weighted Least Square estimator with a Mean- and Variance- adjusted Chi square test statistic) because of the categorical coding categories, showed some support for these two factors through the satisfactory to good factor loadings (>.30; as stated by Tabachnick and Fidell 2007) for all but two items (‘constraining’ and ‘shame inducing’). These two items were not excluded since both constraining (Ballash et al. 2006; Barber 1996) and shame inducing (Barber 1996; Aunola and Nurmi 2004) have been described as essential to psychological control. We decided to stay as close as possible to the theoretical constructs that have been determined in previous studies (Barber 1996; Kunz and Grych 2013). A detailed description of the 16 items measuring maternal warmth and psychological control can be found in the “Appendix”.

Four female research assistants with undergraduate degrees in psychology and previous experience with Specific Affect (SPAFF) coding coded the data. In addition, they underwent a 4-week global coding system conducted by an experienced coding supervisor. The coding manual and example files were reviewed during the first 2 weeks of training. During the final 2 weeks of training, practice files were assigned and calibration meetings were set up. After the training, the research assistants coded the videotapes of this study and rated each item once at the end of the discussions task. Weekly follow-up meetings were set up to minimize coder bias. Twenty percent of the videos were double coded by two research assistants and showed good interrater reliability (ICC = .89).

*Maternal warmth* included the following items: (a) engagement (e.g., asking questions, reminiscing); (b) joint attention (e.g., non-verbal or verbal involvement); (c) balance (e.g., turn-taking); (d) laughter (e.g., joyous laughter); (e) support (e.g., loving statements, concerned questions, reassurance); and (f) validation (e.g., acceptance, paraphrasing). The mean score on all items was used to generate a global score for maternal warmth, with higher scores indicating higher levels of maternal warmth. The reliability for maternal warmth was good (Cronbach’s α = .76).

*Maternal psychological control* included the following items: (a) suggestive questioning (e.g., statement questions); (b) superiority (e.g., pedantic behavior, impose opinion); (c) constraining (e.g., interruption, continuous questioning); (d) invalidation (e.g., denies or argues with statements); (e) criticism (e.g., rejection, attack); (f) intrusiveness (e.g., pervasive talking); (g) shame inducing (e.g., feeling ashamed); (h) guilt inducing (e.g., feeling guilty); (i) provocation (e.g., continuous disagreement, compete); and (j) physics (e.g., physical signs of invalidation). The mean score for all items was used to generate a global score on maternal psychological control, with higher scores indicating higher levels of psychological control. The reliability for maternal psychological control was low (Cronbach’s α = .59).

### Statistical Analyses

First, the means, standard deviations, and correlations among all variables were computed. The association between maternal depressive symptoms, observed mother–child interaction behaviors, and children’s mental health problems was examined using linear regression models with manifest variables in Mplus 6.12 (Muthén and Muthén 1998–2010). The correlation between the observed mother–child interaction behaviors and the interrelations among the three children’s outcome measures were also considered. Furthermore, we used gender of the child, age of the child, and maternal education level as control variables.

Mediation effects were tested in Mplus using the bootstrap method, following the recommendations of Preacher and Hayes (2004). Bootstrapping has the advantage that it does not rely on the assumption that variables are normally distributed, and it can be applied to smaller sample sizes. Furthermore, it does not require meeting the assumptions of significant relations between the independent and outcome variables, which can be the case with small sample sizes due to a lack of statistical power to detect potential present relations (Type II error). Model parameters were estimated with the default estimator of maximum likelihood (ML). Since the model was saturated, goodness-of-fit statistics could not be reported. Instead, the 95 % percentile-based bias corrected and accelerated (BCa) bootstrap confidence intervals were used as a test of significance of the direct and indirect paths. Indirect effects were considered significant when the confidence interval did not include zero. Parameter estimates of the indirect effect were based on 5000 bootstrap samples.

## Results

Table 1 presents descriptive statistics and correlations for all study variables. Mean scores on maternal depressive symptoms indicated that the mothers in the sample could be described as mildly depressed. The percentage of mothers that had a score in the clinical range (a score of 10 or higher on BDI) was 59 %. The scores of children’s internalizing problems on the CBCL ranged from normal to clinical, with a mean score in the normal range. Overall, 32 % of children had a total internalizing score in the clinical range on the CBCL, with 16 % reporting Somatic Problems, 18 % Anxiety Problems, and 35 % Affective Problems. The mean scores of children’s externalizing problems on the CBCL exceeded the clinical cut-off (a T-score of 70 or higher). Furthermore, 71 % of children had a total externalizing score in the clinical range on the CBCL, ranging from 38 % on ADHD to 68 % on Oppositional Defiant Problems and Conduct Problems. The mean score of children’s self-reported depressive symptoms were within the normal range, just below the clinical cut-off (a score of 8 or higher on the SMFQ), with 42 % of children having a score in the clinical range.

**Table 1:** Descriptive statistics and correlations of all study variables

Variable	*M* (*SD*)	Percentage clinical range	1	2	3	4	5	6
1. Maternal depressive symptoms	12.67 (9.64)	59 %	–					
*Mother–child interaction behavior*								
2. Maternal warmth	4.26 (1.06)		−.23*	–				
3. Maternal psychological control	2.29 (0.62)		−.08	−.28**				
*Children’s mental health problems*								
4. Internalizing problems (M)	63.82 (9.39)	32 %	.38***	−.13	.08	–		
5. Externalizing problems (M)	72.01 (5.81)	71 %	.37***	−.09	.18	.60***	–	
6. Depressive symptoms (C)	7.76 (5.75)	42 %	−.03	−.06	.09	.13	.11	–

For informant: M = reported by mother, C = reported by the child

* *p* < .05; ** *p* < .01; *** *p* < .001

Maternal depressive symptoms correlated positively with mother-reported internalizing and externalizing mental health problems of children, but not with children’s self-reported depressive symptoms. Maternal depressive symptoms correlated negatively with maternal warmth but not with psychological control. The observed mother–child interactions did not correlate with mental health problems of the child (neither maternal nor child reports). Moreover, children’s self-reported depressive symptoms did not correlate with maternal reported internalizing or externalizing mental health problems.

**Fig. 1:**
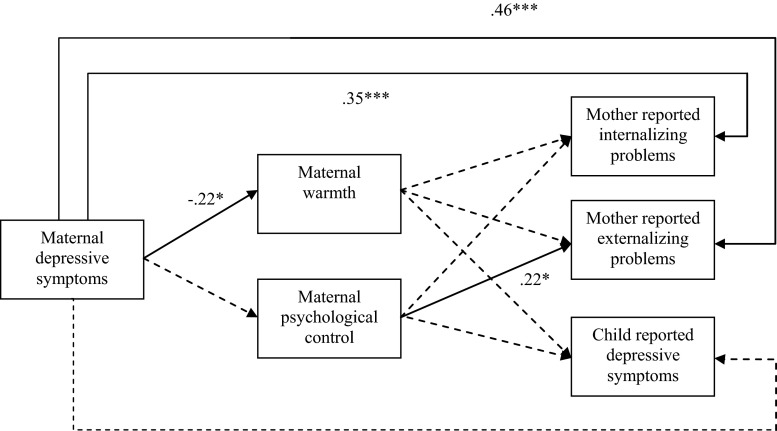
Model of direct paths of maternal mother–child interaction behavior with maternal depressive symptoms and children’s mental health problems. *Black lines* indicate significant paths and *dashed lines* indicate non significant paths. *p < .05, **p < .01, ***p < .001

Figure 1 presents the results of the significant paths of the model while controlling for age and gender of the child and education of the mother. Figure 1 presents the standardized estimates for the significant paths. First, the associations between the independent variable and outcomes were assessed. Maternal depressive symptoms were positively associated with children’s internalizing (*ß* = .35, *p* < .001) and externalizing problems (*ß* = .46, *p* < .001), as reported by the mother, but not with children’s self-reported depressive symptoms, in contrast to our expectations. Next, the direct paths between the independent variables and the mediators were examined. Maternal depressive symptoms were negatively associated with observed maternal warmth (*ß* = −.22, *p* = .02). Unexpectedly, maternal depressive symptoms were not associated with observed maternal psychological control. Regarding the hypothesized direct relations between the mediators and children’s mental health outcomes, as reported by mother and child, we found that observed maternal psychological control was positively related to maternal reports of children’s externalizing problems (*ß* = 0.22, *p* = .01). Observed maternal psychological control was not associated with children’s internalizing problems based on the mother or children’s depressive symptoms, as reported by the child. Unexpectedly, observed maternal warmth was not linked to any of the mental health problems of the child (neither maternal nor child reports). Table 2 shows the standardized path coefficients of the mediation analyses with bootstrapping. The analyses showed that observed maternal warmth and observed maternal psychological control did not mediate any of the relations between maternal depressive symptoms and children’s mental health problems. Models were also run for boys only, which yielded similar results.

**Table 2:** Standardized path coefficients of mediation analyses

	ß	SE	95 % CI^a^
Maternal depressive symptoms to internalizing problems (M)			
Via maternal warmth	0.03	0.02	−0.03, 0.03
Via maternal psychological control	−0.01	0.01	−0.03, 0.01
Maternal depressive symptoms to externalizing problems (M)			
Via maternal warmth	−0.02	0.02	−0.05, 0.02
Via maternal psychological control	−0.02	0.02	−0.06, 0.02
Maternal depressive symptoms to depressive symptoms (C)			
Via maternal warmth	0.01	0.02	−0.03, 0.04
Via maternal psychological control	−0.01	0.02	−0.04, 0.02

For informant: M = reported by mother, C = reported by the child

^a^Bias corrected and accelerated (BCa) bootstrap confidence intervals

## Discussion

The first objective was to examine whether maternal depressive symptoms were positively related to children’s mental health problems. Consistent with our premise and previous studies (e.g., Turney 2011), we found mothers with higher levels of depressive symptoms reporting more internalizing and externalizing mental health problems in their children. Contrary to our expectations, children of mothers with higher levels of depressive symptoms did not report more depressive symptoms. Thus, from the mother’s perspective, we found a relation between maternal depression and child psychopathology, but this relation was not present when using child report. It has been suggested that maternal depressive symptoms have a great influence on informant discrepancies in reporting children’s mental health problems (Fergusson et al. 1993; Kraemer et al. 2003). In general, two theories dominate the field of research on the effect of maternal depressive symptoms on maternal reports of children’s mental health. While the depression distortion bias hypothesis argues that depressed mothers over-report problems in their children due to their ‘depressive schema’ (Richters 1992), the competing accuracy theory claims that depressed mothers are accurate reporters due to their heightened awareness of potential problems in their children (Fergusson et al. 1993). The results of this study appear to provide more evidence for the ‘distortion bias’ hypothesis, suggesting that depressive cognitions and perspectives of the mother have a greater (negative) effect on their reports of their child’s mental health compared to the child’s experiences of their own mental well-being. Another possibility is that children with externalizing problems have more difficulties recognizing, reporting, or admitting depressive symptoms. Boys are more likely to express emotional problems in ‘acting out behavior’, whereas girls are more likely to express emotional problems through internalizing symptoms (Chaplin and Aldao 2013). Observers, but also boys themselves who may overly attend to angry feelings instead of sad ones, may not interpret acting out behavior as a symptom of depressive symptoms. Although there is no ‘gold standard’ to determine which informant is more accurate, our findings emphasize the importance of assessing problems from multiple informants’ perspectives and suggest that future studies might need to consider other more objective measurements of children’s mental health problems, such as clinical observations.

Our second purpose was to study the mediating effect of the observed mother–child interaction behaviors on the relation between maternal depressive symptoms and children’s mental health problems. Contrary to our expectations, we found no mediating effect of maternal warmth and psychological control on the relation between maternal depressive symptoms and children’s mental health problems. Several explanations can account for this finding. First, it is possible that the mediating effect of mother child interactions on the relation between maternal depressive symptoms and children’s mental health problems is age specific. In younger children, interactions, such as maternal sensitivity and responsiveness, might exert a stronger mediating effect because these behaviors are shown to be essential in forming secure attachment and internal working models (Egeland and Farber 1984). Later during adolescence, mother child interactions undergo major transitions (Granic et al. 2003), which might intensify the effect of these interactions (Pugh and Farrell 2012). The current study investigated a middle childhood sample (age 8–12 years), a rather stable developmental period compared to toddlerhood and adolescence. It might be that interactional behaviors have greater predictive power during developmental periods when interactions are still in their formation phase (toddlerhood) or are in a phase of critical alterations (adolescence), but they are of less relevance during more consolidated developmental periods. Extensive longitudinal studies would be needed in order to investigate these hypotheses.

Second, an explanation of this null finding might be found in the specific nature of our sample. Previous studies that reported a mediating effect were all conducted with community samples (Burt et al. 2005; Karazsia and Wildman 2009; Kiernan and Huerta 2008; Pugh and Farrell 2012). Our study investigated a sample of clinically aggressive children *and* mothers scoring high on depression symptoms (59 % scored in the clinical range and the mean score of the mothers was within the mildly depressed range). This might imply that our findings are specific to mothers with high levels of depressive symptoms and clinically aggressive children. It is known that aggressive children elicit certain maternal interactions and that children’s aggression contributes to maternal depressive symptoms. Multiple longitudinal studies have demonstrated a bidirectional relation between maternal depressive symptoms and children’s aggression (e.g., Raposa et al. 2011). The coercion theory (Patterson 2002) postulates that aggressive children and their parents mutually reinforce each other’s (negative) behavior. When the child behaves aggressively, the parent demands obedience. The child returns with an escalation of aversive behavior, and the parent with an escalated attempt of discipline. Eventually the parent gives in, which reinforces the child’s aggressive behavior and at the same time, leads to maternal feelings of hopelessness regarding her ability to discipline the child (Fite et al. 2006). This might lead to elevated levels of stress (Raposa et al. 2011) and increased depressive symptoms.

Third, the current study focused on two specific aspects of mother–child interactions, maternal warmth and psychological control, using a global observation system. Thus, the conclusion that mother–child interactions *in general* do not mediate the relation between maternal depressive symptoms and mental health problems in children is not warranted. It is possible that a) other relevant constructs, such as behavioral control (e.g., depressive mothers being more permissive, *see* Topham et al. 2010) and self-regulation (e.g., children with warm mothers show better regulation skills compared to rejecting and controlling mothers, *see* Baker and Hoerger 2012), or b) more structural aspects of the interaction (e.g., rigidity in parent–child interaction, *see* Granic et al. 2003) mediate this relation. Further research would be needed to investigate these hypotheses.

With regard to our direct path analyses, we found that mothers with more depressive symptoms showed less warmth in interaction with their children, which was consistent with our expectations and prior observational research (e.g., Feldman 2010; *meta*-*analysis of* Lovejoy et al. 2000). Furthermore, we found that mothers who exerted greater psychological control while interacting with their child reported more externalizing mental health problems in their child. This is in contrast with numerous studies on adolescents, which have found a relation between psychological control and *internalizing* mental health problems (Barber et al. 2005; Soenens et al. 2008). Childhood studies on psychological control and children’s mental health problems are relatively scarce; however, their results suggest that maternal psychological control is linked to relational and physical aggression (Casas et al. 2006) and to externalizing mental health problems (Verhoeven et al. 2010). Again, this implies, as noted earlier, that the investigated associations might be age specific, that the relation between maternal psychological control and children’s mental health problems depends on the age of the child, and that our results are specific to middle school children.

The presented results and theoretical explanations of our study should be placed within a context of several methodological limitations that warrant cautiousness in generalizing these findings. First, the data in this study were cross-sectional, which prevented us from examining true mediation effects. Mediation analyses require temporal sequencing from maternal depression to children’s mental health problems through mother–child interaction behavior (MacKinnon et al. 2007). Since all measurements have been conducted at the same point in time, this violates the temporal precedence. Longitudinal or intervention designs need to be considered in future studies on mother–child interaction behavior. Second, the reliability of observed maternal psychological control was poor (α = .59), so this construct should be interpreted with caution. However, Cronbach’s α has recently been disputed because the assumptions (e.g., unidimensionality and uncorrelated errors) underlying this measure are often not met. It is mostly used as an indicator of internal consistency while in fact it is based on the degree of interrelatedness of items and has little to do with the actual internal structure of a test (Sijtsma 2009). This means that although it is common to report alpha measures, low alpha values do not imply that the construct is not adequate.

Third, while the reported direct effects between maternal depressive symptoms and mother–child interaction behavior were significant and provide further evidence for the complex dynamics underlying the mechanism in the transmission of risk from maternal depressive symptoms to children’s mental health problems, many of the effects were small, which might be due to the presence of other contributing factors. Moreover, the sample size was rather small to conduct the mediation analyses. In fact, a post hoc power analyses, based on effect sizes from previous studies (Lovejoy et al. 2000; McLeod et al. 2007), revealed that we had reasonable power to detect an effect of maternal psychological control but that the study was underpowered to detect an effect of maternal warmth (MacKinnon et al. 2007). Thus, the results need to be interpreted with caution and more research with bigger sample sizes are needed. If several different mother–child interaction factors are investigated simultaneously it is important to base the power calculation on the smallest effect to guarantee sufficient power for all paths in the model.

Furthermore, we did not include children’s self-reported externalizing problems in the study, since parents are assumed to report externalizing problems more accurately compared to children (Kerr et al. 2007). Consequently, we could not determine whether our findings regarding externalizing problems are due to the typical informant disagreement between mother and child (De Los Reyes 2011). Finally, this study focused solely on the role of the mother. Recently, the role of the father in the transmission of risk has received increased attention. For example, Kane and Garber (2009) found that father-child conflict mediated the relations between paternal depressive symptoms and children’s externalizing problems, even above the effect of maternal depressive symptoms. Clearly, future research based on the influence of both parents and/or other involved caregivers is needed.

Despite the limitations, this study is the first to integrate observations of mother–child interactions with both maternal and child reports to study their effects on children’s mental health problems. It showed an absence of mediating effects of observed mother–child interactions, regardless of the strong direct path between maternal depressive symptoms and mother-reported children’s mental health problems. Furthermore, our study showed no relation between maternal depressive symptoms and child self-reported depressive symptoms. These results underline the complexity of the process of risk transmission from depressive symptoms in mothers to mental health problems in their children and emphasize the need to use a multi-informant and multi-method approach to assess children’s mental health problems for both research and clinical purposes.

## Appendix

Description of the items measuring maternal warmth and maternal psychological control.

**Table:**

Warmth	
Engagement	Mom seems to be ‘in tune’ with her child. She is interested and support his or her ideas, e.g., asking questions, active listening, shared humor, inside jokes, reminiscing
Joint attention	There is a shared focus of attention between mother and her child. Mom shows verbal and/or non-verbal activity indicating involvement (e.g., making eye contact, slight nodding)
Balance	There is a balanced conversational style of interaction between mother and her child. There is relevant turn-taking where the comments or questions of one follow from the utterances of the partner and the participation of the discussion is equal
Laughter	Mom shows joyous laughter. This does not enhance nervous laughter (a forced laughter that often does not ‘fit in’ with the context of the conversation, there was nothing funny)
Support	Mom shows direct expressions of caring or comfort (e.g., loving/caring statements, concerned questions/statements, joy, compliments, general support, empathy, reassurance/comfort, physical touch). Her voice is neutral or ‘up’
Validation	Mom shows respect in her communication. She is accepting and open to suggestions, even if the child’s feelings or ideas are at odds with her own (e.g., active listening, understanding/acceptance, paraphrasing, apology, finishing sentences)
Psychological control	
Suggestive questioning	Mom asks a questions, which is actually a statement, e.g., Questions starting with “Wouldn’t you…/Couldn’t you…/Shouldn’t you…”
Superiority	Mom talks to the child in a pedantic manner, e.g., she uses the statements “it’s the way it is” of “everybody does…” in order to ground her opinion
Constraining	Mom interrupts the child or mom does not allow the child to express his or her opinion, e.g., mom asks a question, but does not allow the child to answer by continuing to talk or answer the question for the child
Invalidation	Mom validates the behavior or opinion of the child as wrong, e.g., she denies or argues with statements of the child
Criticism	Mom comments the behavior or expressions of the child, e.g., she rejects the opinion of the child or personally attacks the child
Intrusiveness	Mom comes physically close to the child and/or talks in a pervasive manner, e.g., she holds the head of the child in order to let the child make eye contact
Shame inducing	Mom lets the child feels ashamed of his or her behavior, expression/opinion, and/or general circumstances. This often concerns something that has happened in the past
Guilt inducing	Mom lets the child feels guilty of his or her behavior, expression/opinion, and/or general circumstances. The emphasize lies on the responsibility of the child for the behavior, expression/opinion, and/or general circumstances
Provocation	Mom disagrees continually with the child, independent of the context. Mom reacts negatively on everything the child says, e.g., asking ironic questions or mom competes with the child
Physics	Mom shows physical signs of invalidation, such as rolling with her eyes
